# Thermal Scanning-Probe Lithography for Broad-Band On-Demand Plasmonic Nanostructures on Transparent Substrates

**DOI:** 10.1021/acsanm.3c04398

**Published:** 2023-10-03

**Authors:** Lorenzo Ramò, Maria Caterina Giordano, Giulio Ferrando, Paolo Canepa, Francesca Telesio, Luca Repetto, Francesco Buatier de Mongeot, Maurizio Canepa, Francesco Bisio

**Affiliations:** † OptMatLab, Dipartimento di Fisica, Università di Genova, Via Dodecaneso 33, I-16146 Genova, Italy; ‡ LabNano, Dipartimento di Fisica, Università di Genova, Via Dodecaneso 33, I-16146 Genova, Italy; § Dipartimento di Fisica, Università di Genova, Via Dodecaneso 33, I-16146 Genova, Italy; ∥ Nanomed Laboratories, Dipartimento di Fisica, Università di Genova, Via Dodecaneso 33, I-16146 Genova, Italy; ⊥ CNR-SPIN, C.so Perrone 24, I-16152 Genova, Italy

**Keywords:** scanning-probe lithography, noninvasive nanolithography, nanophotonics, plasmonic nanoantennas, optical microspectroscopy, transparent conductive oxides

## Abstract

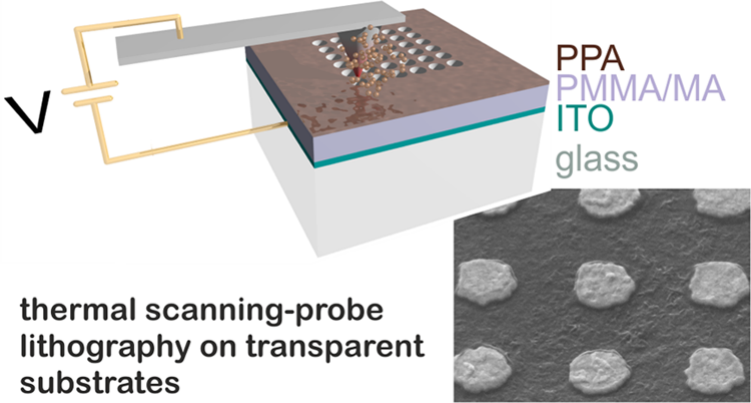

Thermal scanning-probe lithography (t-SPL) is a high-resolution nanolithography technique that enables the nanopatterning of thermosensitive materials by means of a heated silicon tip. It does not require alignment markers and gives the possibility to assess the morphology of the sample in a noninvasive way before, during, and after the patterning. In order to exploit t-SPL at its peak performances, the writing process requires applying an electric bias between the scanning hot tip and the sample, thereby restricting its application to conductive, optically opaque, substrates. In this work, we show a t-SPL-based method, enabling the noninvasive high-resolution nanolithography of photonic nanostructures onto optically transparent substrates across a broad-band visible and near-infrared spectral range. This was possible by intercalating an ultrathin transparent conductive oxide film between the dielectric substrate and the sacrificial patterning layer. This way, nanolithography performances comparable with those typically observed on conventional semiconductor substrates are achieved without significant changes of the optical response of the final sample. We validated this innovative nanolithography approach by engineering periodic arrays of plasmonic nanoantennas and showing the capability to tune their plasmonic response over a broad-band visible and near-infrared spectral range. The optical properties of the obtained systems make them promising candidates for the fabrication of hybrid plasmonic metasurfaces supported onto fragile low-dimensional materials, thus enabling a variety of applications in nanophotonics, sensing, and thermoplasmonics.

## Introduction

The fabrication of next-generation hybrid nanodevices, such as those combining complex contacts and interconnects^1−9^ or tailored nanoantennas with two-dimensional (2D) materials, is under intense scrutiny for optoelectronic, sensing, and photoconversion applications.^10−17^ There is particular interest in novel nanolithographic approaches that can address nanofabrication on transparent substrates while avoiding the use of prealigned markers and energetic beams (such as those employed in electron-beam lithography or focused ion-beam lithography), which are prone to damaging sensitive low-dimensional materials.^18−20^

Thermal scanning-probe lithography (t-SPL) is a recently developed direct-writing mask-less scanning-probe technique that is progressively gaining more interest thanks to the fact that its nanometric resolution can be exploited both for the deterministic writing of nanopatterns and for the thermal probing of thin films or fragile nanosystems at the nanoscale.^21−23^ This is possible thanks to the noninvasive thermal probe–sample interaction that ensures the integrity of the sample unlike other techniques of comparable resolution.^24−29^ Taking advantage of these unique features, t-SPL has been already largely exploited for the nanopatterning of fragile 2D materials.^30−33^ Since the high-resolution nanolithography is naturally accompanied by the in situ contact mode imaging, the t-SPL technique uniquely enables the real-time imaging of the thermally defined nanostructures. Additionally, the precise alignment of deterministic nanostructures with respect to target features can be achieved thanks to a closed-loop configuration of the system.^34^ This way, cumbersome and time-consuming alignment processes can be avoided to fabricate nanodevices and/or decorating nanostructures on top of pre-existing nanomaterials.^35^ This method is also well suited for 3D nanopatterning since it is possible to achieve a subnanometric resolution in the z direction.^36−41^

In t-SPL, a nanosized silicon tip is integrated into a special cantilever equipped with an ad hoc electric circuit, which can increase the tip temperature in a controlled way;^42^ the final result is a nanometric hot stylus that can impress a pattern on thermosensitive materials.^43,44^ Typically, one or more layers of thermolabile materials are deposited on top of a substrate and the interaction between the hot tip and the deposited thin films is exploited to impress a nanopattern, which can be adopted as a mold or as an etching mask.^45−48^

For lithographic purposes, it is possible to sublimate special thermoresists, like polyphthalaldehyde (PPA), which degrades into volatile monomers above a certain temperature.^49^ The lithographic resolution is determined by the spatial extension of the thermo-chemical–mechanical alteration of the patterned material (which can be as low as few tens of nanometers thanks to the sharp silicon tip).^50^ An alternative approach, recently investigated, exploits a nanoscopic AFM tip to precisely align block copolymer domains with nanometric resolution, avoiding the degradation of the polymeric layer.^51^

Accurate control on the tip position and in turn on spatial resolution during the hot-tip writing process is enabled by the capacitive sensor/actuator formed by the cantilever in proximity to the conductive substrate. This approach thus fits perfectly with conducting and semiconducting substrates, enabling high-resolution nanopatterning capabilities. However, there is a crucial deal with the possibility of accurately controlling the t-SPL onto optically transparent dielectric substrates that represent state-of-the-art templates for optoelectronic, nanophotonic, and sensing applications. In this work, we report a novel method for the noninvasive, high-resolution nanolithography of arrays of laterally disconnected nanostructures onto optically transparent substrates. We show that depositing an ultrathin layer of transparent conductive oxide (TCO) on top of the insulating substrate is sufficient to recreate the ideal capacitive coupling required for high-resolution tip actuation, all while preserving the substrate transparency.

Previously published reports already highlighted the importance of t-SPL for developing contacts and interconnects to 2D-TMD-based devices and, in particular, described clearly the possibility to directly overlay the lithographic pattern on top of the ultrathin material^52,53^ without using prealigned grids and energetic probes. Here, we instead focus our attention on a novel nanofabrication route that extends the current limitations of t-SPL-based approaches for nanophotonic applications. More in detail, we test this method for the deterministic nanofabrication of periodic arrays based on plasmonic gold, engineered in shape and size in order to control their optical response over a broad-band spectral range. By means of transmittance microspectroscopy, we show the capability to tune localized surface plasmon resonances (LSPRs) in the nanoarrays over the visible and near-infrared spectra, demonstrating the impact of this platform for various applications in nanophotonics and sensing.^54−58^

## Materials and Methods

### Substrate Preparation

A soda-lime glass (1 × 1 × 0.2 cm^3^) is rinsed for 10 min in both acetone and isopropylic alcohol and loaded in a custom-made vacuum chamber with a base pressure on the order of 10^–6^ mbar. A thin film of indium–tin oxide (ITO) is deposited by means of radio frequency (RF) sputtering at a power of 10 W, with a flux of 4.6 Å/s. The typical thickness of the ITO layer, measured by spectroscopic ellipsometry, is (10 ± 1) nm (J.A. Woollam M2000 variable-angle ellipsometer). The *rms* roughness, assessed by atomic force microscopy (AFM), is around 2 nm. In order to ensure the electric connection between the scanning head of the NanoFrazor and the sample, an electric-grade copper wire coated with kapton is glued on the sample using silver paste. One end of the copper wire is electrically connected with the ITO layer, while the second one (clamped with a plastic screw on the NanoFrazor sample holder) closes the electric circuit.

A 95 nm-thick copolymer layer based on polymethyl-methacrylate and methacrylic acid (PMMA/MA) is deposited via spin coating using a solution of AR-P617 from Allresist (6000 rpm for 60 s with the spin coater Laurell WS-650MZ-23NPPB) and cured for 90 s at 180 °C. The second layer of resist, made of PPA and with a thickness of 25 nm, is deposited via spin coating a solution of AR-P8100.06, solid content: 5.5 wt % in anisole from Allresist (6500 rpm for 60 s with the same spin coater) and cured for 60 s at 110 °C.

When the required lateral size of the nanostructures drops below 150 nm, the thickness of the PMMA/MA has to be reduced to 40 nm by diluting the PMMA/MA solution with the thinner AR 600-07 from Allresists (propylene glycol monomethyl ether) in order to reach a solid content of about 1.5 wt %. This diluted solution is spin-coated at 4000 rpm for 60 s and cured for 90 s at 180 °C. The reduced thickness of the transfer layer helps decrease the risk of collapse of high-resolution patterned structures during the development process.

### Thermal Scanning-Probe Lithography

Patterning via t-SPL of the thin thermosensitive PPA layers was performed with a NanoFrazor Scholar from Heidelberg Instruments GmbH to realize high-resolution arrays of nanoantennas supporting localized surface plasmon resonances (LSPRs) tunable across a broad-band spectral region from the visible to the near-infrared.

### Development, Material Deposition, and Lift-Off

A solution of 5% (v/v) of deionized water in isopropylic alcohol was used as the etcher of the PMMA/MA layer. A calibration of the etching rate was performed by acquiring AFM images of the patterns at different etching times in order to follow the development process step by step. The development was considered completed after a total etching time of 150 s for structures bigger than 150 nm and 60 s for structures smaller than 150 nm (i.e., with a reduced thickness of the PMMA/MA layer). After each immersion in the etching solution, the sample was rinsed in isopropylic alcohol and then dried with a nitrogen flow.

The sample was then loaded in a custom-made ultrahigh vacuum chamber with a base pressure on the order of 10^–9^ mbar. In the chamber, Au films with a Cr adhesion layer were deposited by molecular beam epitaxy (MBE). For structures bigger than 150 nm, the thickness of Au and Cr was 18 and 3 nm, respectively, while for smaller structures, the thickness of the two metals was reduced to 1.5 nm for Cr and 13.5 nm for Au in order to match the corresponding decrease of the (PMMA/MA)/PPA resist layer.

The lift-off process started by soaking the sample in agitated acetone (the resist solvent) for 2 h at room temperature. The resist was removed by generating turbulence in acetone with a syringe and then sonicating for 15 min. The sample was then extracted from acetone, rinsed in isopropylic alcohol, and dried with a nitrogen flow.

### Sample Morphology

The sample morphology after fabrication was analyzed by means of AFM and scanning electron microscopy (SEM). AFM topographies were acquired using a multimode/nanoscope V system (Bruker, Germany). The instrument was operated in tapping mode using silicon cantilevers (OMCL-AC160TS, Olympus, Japan) with a typical resonance frequency of about 300 kHz. The nominal tip radius was 7 nm. The images were analyzed using the open source software Gwyddion.^59^

SEM was carried out, acquiring secondary electrons, with two instruments: a field emission microscope (CrossBeam 1540xb, Carl Zeiss, Germany) using an in-lens detector and a conventional tungsten emitter microscope (SU3500, Hitachi, Japan). The accelerating voltage was set at 20 and 10 kV, respectively.

### Microtransmittance Spectroscopy

Transmittance spectra are obtained by means of a homebuilt microspectroscopic setup.^60^ For this experiment, two spectrometers are employed in tandem (AvaSpec-ULS2048XL-EVO and AvaSpec-NIR256-2.5-HSC-EVO from Avantes B. V., NS Apeldoorn, The Netherlands), covering a spectral range from 400 to 2240 nm with a measurement spot size of approximately 20 μm diameter.

## Results and Discussion

**Figure 1:**
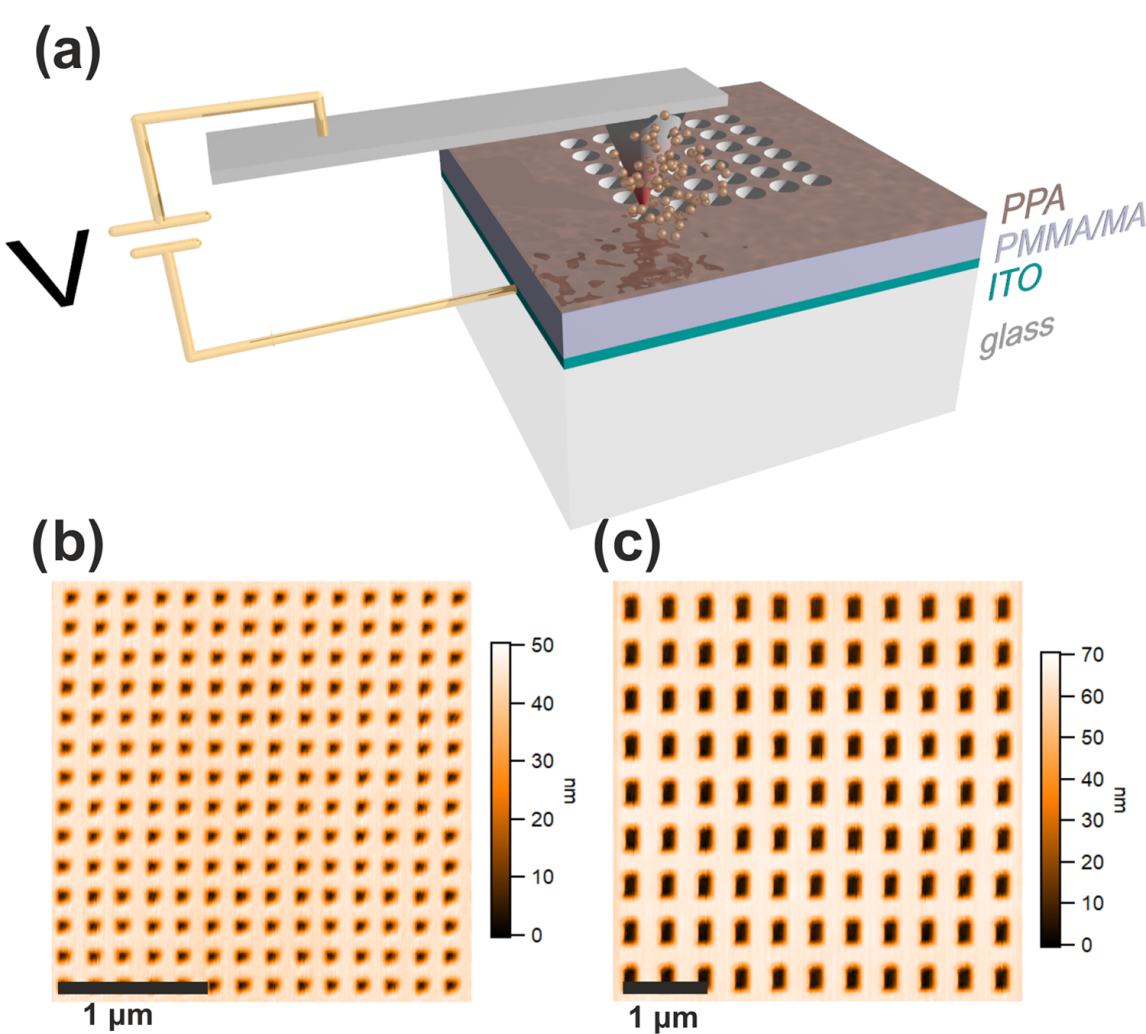
(a) Sketch of the high-resolution t-SPL lithography patterning configuration on transparent substrates. The heated scanning head of the t-SPL instrument is electrically connected with the TCO layer deposited between the substrate and the polymeric thin films. (b) and (c) t-SPL patterns imaged in situ using AFM capability of the t-SPL tool of an array of holes with a diameter of 50 nm and a gap of 150 nm (ND1) and an array of elongated cavities with dimensions 150 nm × 250 nm and gap of 300 nm (E1), respectively.

The lithographic configuration adopted is sketched in Figure 1a; there, we highlight that, thanks to the TCO ultrathin film it is possible to precisely control the nanolithography onto a thermosensitive material, obtaining nanostructures of arbitrarily defined shape, with high spatial resolution, at the surface of a transparent insulating substrate.

We fabricated nanostructure arrays with a square arrangement and nominal geometries as reported in Table 1.

**Table 1:** Nominal Geometries of the Fabricated Arrays of Nanostructures

sample	morphology	size/gap
ND1	nanodiscs	⌀ 50 nm/gap 150 nm
ND2	nanodiscs	⌀ 100 nm/gap 300 nm
ND3	nanodiscs	⌀ 100 nm/gap 200 nm
ND4	nanodiscs	⌀ 200 nm/gap 400 nm
ND5	nanodiscs	⌀ 300 nm/gap 400 nm
E1	rectangle	150 × 250 nm/gap 300 nm

We opted for nanodiscs since their plasmonic response is isotropic and simpler than other shapes, enabling us to show its tunability by changing the diameter and the gap between nanodiscs in order to cover a broad-band spectral range in an on-demand fashion. Elongated nanostructures were fabricated as well to show that this technique has no limits regarding bidimensional shapes and to introduce a further parameter in the spectral tuning of the plasmonic response. Figure 1b,c shows the t-SPL patterns of the structures ND1 and E1, respectively, imaged in situ and in operando with the t-SPL tool exploited as AFM.

**Figure 2:**
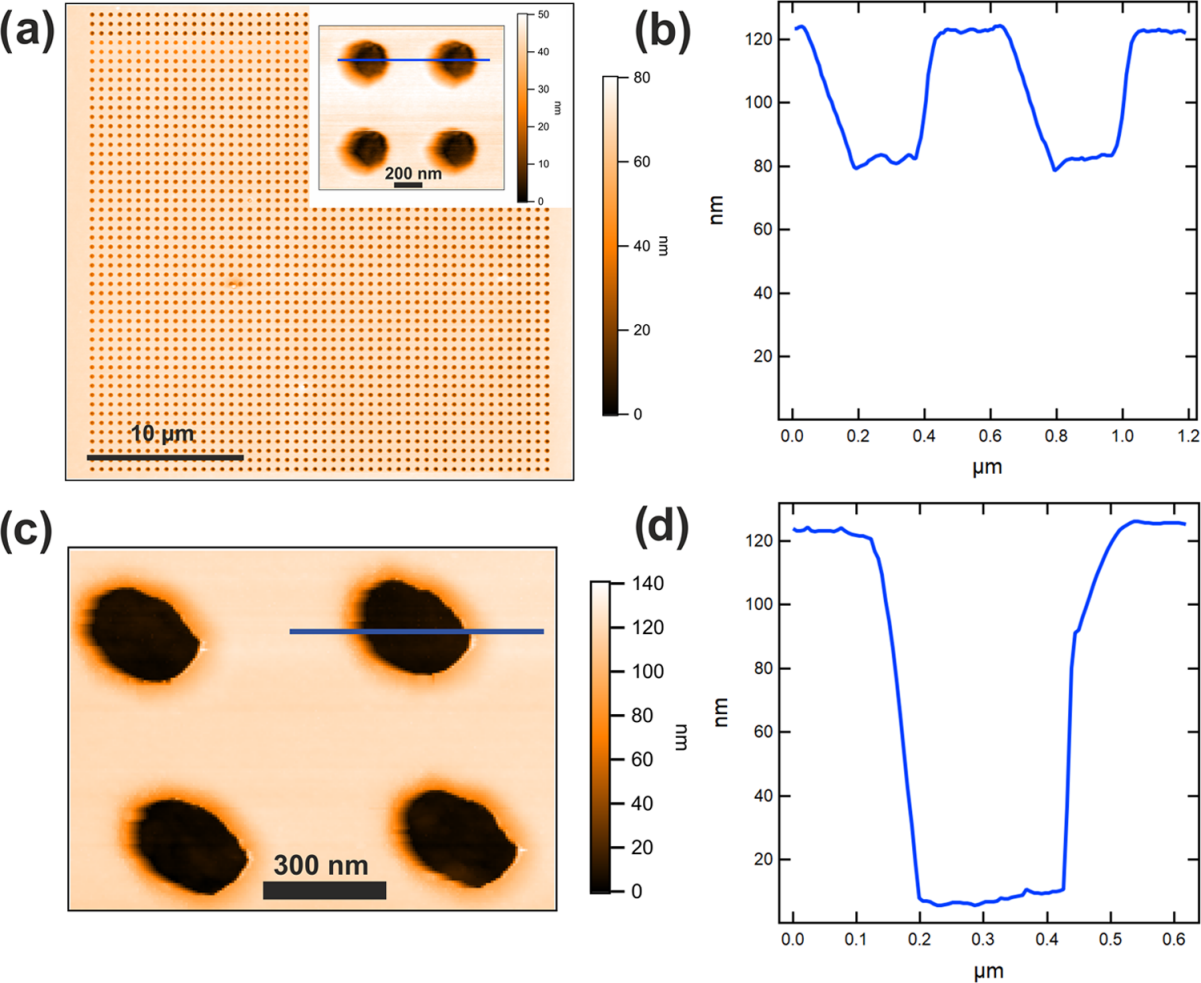
(a) t-SPL pattern of an array of holes with a diameter of 200 nm and a gap of 400 nm nominally over an area of approximately 30 μm × 30 μm (ND4) imaged in situ using AFM capability of the t-SPL tool. (b) Depth profile of the impressed pattern along the blue line of the (a) inset. (c) AFM image of a portion of the same pattern after 150 s of development in the etching solution made of 5% (v/v) deionized water in isopropylic alcohol. (d) Depth profile corresponding to the blue line in panel (c).

Figure 2a shows the ND4 pattern impressed on the resist, exploiting the AFM functionality of the t-SPL instrument. On the top-right part of the image is a zoomed-in view of the pattern, which contains only four holes. Figure 2b shows the depth profile of the impressed pattern along the blue line in the inset of Figure 2a. The average depth of the patterned regions is about 40 nm, and since this value is higher than the thickness of the PPA layer (25 nm), the pattern can be effectively transferred through the PMMA/MA layer during the wet etching process.

Figure 2c shows an AFM image of a portion of pattern ND4 after 150 s of development in the etching solution made of 5% (v/v) deionized water in isopropylic alcohol; Figure 2d reports the depth profile corresponding to the blue line in panel c, drawn across a hole. The average depth of the holes after etching the PMMA/MA copolymer layer is close to 115 nm, which is compatible with the total thickness of the double layer of resist. This value indicates that at the bottom of the hole the substrate under resist was exposed and that the etching process was completed successfully. At this stage, the polymer lithographic mask has been employed for confining the deposition of Au nanostructures with a thickness of 18 nm.

**Figure 3:**
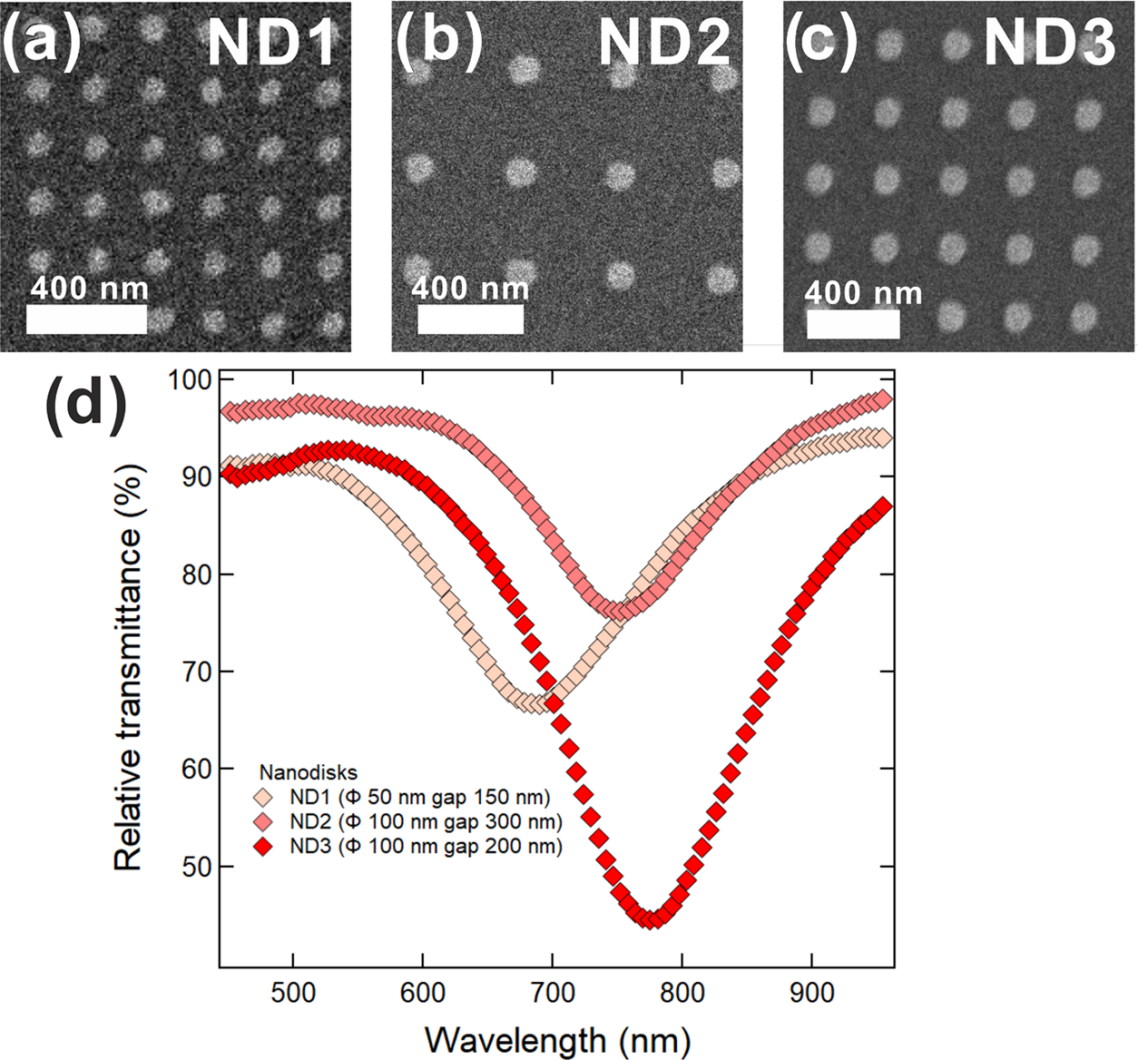
Results of the fabrication of structures smaller than 150 nm obtained via t-SPL on glass covered by ITO. (a) SEM micrograph of the array of nanodiscs with a nominal size of 50 nm for the diameter and 150 nm for the gap (ND1) (SU3500, Hitachi, accelerating voltage 10 kV), (b) SEM micrograph of the array of nanodiscs with a nominal size of 100 nm for the diameter and 300 nm for the gap (ND2) (SU3500, Hitachi, accelerating voltage 10 kV), (c) SEM micrograph of the array of nanodiscs with a nominal size of 100 nm for the diameter and 200 nm for the gap (ND3) (SU3500, Hitachi, accelerating voltage 10 kV). (d) Relative transmittance spectra of the arrays of nanostructures ND1, ND2, and ND3.

The effective morphology of the nanostructures obtained after Au/Cr deposition and lift-off of the polymer mask was assessed via SEM imaging. Figure 3 shows the results for the fabrication of nanostructures smaller than 150 nm (ND1, ND2, ND3). Panels (a)–(c) show, respectively, the SEM micrograph of arrays ND1, ND2, and ND3.

Figure 3d shows the transmittance spectra of arrays ND1, ND2, and ND3 normalized with respect to the bare substrate. All of the spectra in this graph exhibit a single marked absorption dip corresponding to the excitation of the dipolar mode of the LSPR. The absorbance of the structures depends on the Au thickness, the size of the individual nanostructures, and their density. Therefore, ND3 shows a more pronounced dip in transmission than ND2 because reducing the gap between the nanostructures, their density (and so the metal coverage) increases. As a general trend, we can consider that for a fixed thickness h of the Au nanodisc and increasing the diameter *D*, the LSPR resonant wavelength red-shifts when the ratio *D*/*h* increases.^61^ An additional red shift is observed for a constant diameter when the gap separating the nanodiscs is reduced like, e.g., ND2 and ND3, since interparticle coupling increases.

**Figure 4:**
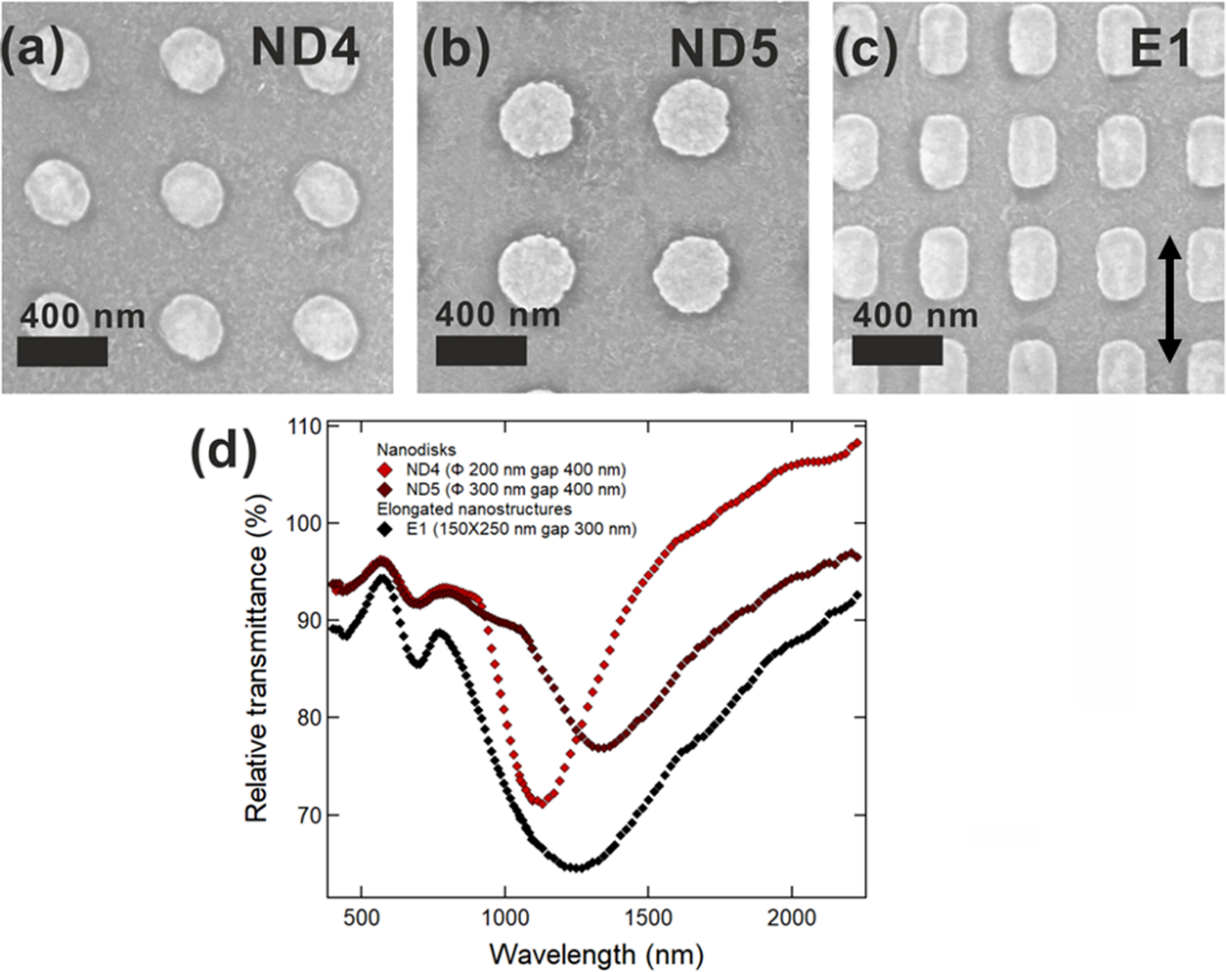
Results of the fabrication of structures larger than 150 nm obtained via t-SPL on glass covered by ITO. (a) SEM micrographs of circular nanostructures with a nominal diameter of 200 nm and a nominal gap of 400 nm (ND4) (CrossBeam 1540xb, Carl Zeiss, in-lens detector, accelerating voltage 20 kV, normal incidence), (b) SEM micrographs of circular nanostructures with a nominal diameter of 300 nm and a nominal gap of 400 nm (ND5) (CrossBeam 1540xb, Carl Zeiss, in-lens detector, accelerating voltage 20 kV, normal incidence), (c) SEM micrographs of elongated nanostructures with a nominal size of 150 nm × 250 nm and a gap of 300 nm (E1) (CrossBeam 1540xb, Carl Zeiss, in-lens detector, accelerating voltage 20 kV, normal incidence; the arrow indicates the electric field orientation for the optical measurements), and (d) relative transmittance spectra of the nanostructures ND4, ND5, and E1.

Figure 4 shows the results for the fabrication of nanostructures bigger than 150 nm. Panels (a)–(c) show, respectively, the SEM micrograph of arrays ND4, ND5, and E1. Figure 4d shows the relative transmittance spectra of the nanostructures ND4, ND5, and E1. Each spectrum is characterized by two transmittance dips: a more intense one at higher wavelengths and a less intense one at shorter wavelengths. The first one is the LSPR dipolar mode, while the second one is due to the excitation of LSPR multipolar modes; for the elongated nanostructures (E1), we can exclude the excitation of the transverse dipolar mode since the polarization of the impinging light is parallel to the longitudinal axis of the nanostructures.

Our results, obtained with plasmonic nanostructures prepared by t-SPL lithography, are in close match with experiments obtained by conventional lithography and with numerical simulations of the optical response available in refs (62−66). Among the broad literature reporting optical simulations for plasmonic nanodisc arrays, we can, e.g., refer to the finite element simulations of ref (67), which consider Au nanodisc arrays of comparable geometry, prepared by conventional interference lithography. As a general rule, the comparison is semiquantitative and affected by details such as the dielectric environment surrounding the Au nanodisc (leading to an increase in the resonant wavelength for increasing refractive index), the thickness of the Au film (which produces a more substantial red shift when the thickness is reduced below 10 nm), and the gap separating the nanodiscs (which results in a red shift of the plasmon frequency, more substantial at smaller separations below 50 nm due to near-field coupling^63^). The simulations of ref (67) highlight the presence of fundamental dipolar plasmon modes, which red-shift from about *l* = 600–1200 nm with increasing diameter *D* from 50 to 300 nm, in the case of 10 nm thick Au discs, in qualitative agreement with the experimental extinction spectra of Figures 3 and 4, which show a red shift of the plasmon resonance for samples ND1, ND2, ND4, and ND5 with increasing nanodisc diameter. Also to be highlighted in Figure 3c is the red shift of the plasmon resonance of samples N2 and ND3, at a fixed nanodisc diameter, when the gap is reduced from 300 to 200 nm.

The simulations of ref (67) also evidence the appearance of higher-order multipolar modes for nanodisc diameters approximately above 300 nm. Such results are in good agreement with the data shown in Figure 4d for the larger nanodisc ND5, which has a fundamental dipolar mode of around 1300 nm and the higher-order mode at a halved wavelength of 650 nm.

Finally, if we consider anisotropic nanorods such as those of sample E1 in Figure 4, a geometric shift of the resonance is expected, depending on the polarization axis of the illuminating radiation. As a general rule (see, e.g., ref (62)), a red shift of the plasmon resonance is expected when polarization is parallel to the nanorod long axis and a red shift is observed for the increasing nanorod length at a fixed width.

From the SEM analysis, it is possible to assess the effective morphology and the extent of the enlargement of the nanostructures with respect to nominal dimensions; this information is reported in Table 2. SEM micrographs also show in-plane and line-edge roughnesses of the nanostructures. The first is due both to the effects of the ITO roughness and to the metal grain size, while for the second, there is also a contribution due to the finite size of the scanning tip. The entity of this deviation from the ideal shape is in the order of a few nanometers, thus not causing any significant alteration in the optical response of the nanoparticle arrays.

**Table 2:** Effective Geometries of the Fabricated Arrays of Nanostructures

sample	effective size/gap (nm)	areal enlargement (%)
ND1	⌀ 60 ± 5/gap 125 ± 5	44
ND2	⌀ 103 ± 4/gap 288 ± 7	6
ND3	⌀ 115 ± 5/gap 170 ± 10	32
ND4	⌀ 249 ± 7/gap 301 ± 9	55
ND5	⌀ 323 ± 3/gap 353 ± 8	16
E1	196 ± 5 × 306 ± 4/gap 181 ± 5	60

Knowing the entity of these geometrical alterations helps to increase the precision of future fabrications by properly reducing the size of the structures in the mask. Possible strategies for limiting the nanostructure broadening with respect to nominal dimensions can be the employment of a thinner polymeric layer and the use of sharper heating tips in order to limit the broadening in the writing stage.

The LSPRs of the obtained nanostructures, in particular, those smaller than 150 nm, exhibit good-quality factors (ratio between the intensity of absorption and full width at half-maximum of the peaks), which indicate the reduced polydispersity in size of the structures and the good quality of the fabrication process, making these arrays promising for plasmonic and thermoplasmonic applications. Remarkably, this nanolithography can be applied in a noninvasive way when fragile low-dimensional materials lying on transparent insulating substrates need to be decorated by plasmonic nanoantennas, thus opening new possibilities in nanophotonics and sensing.

## Conclusions

In this work, we demonstrate the noninvasive thermal scanning-probe nanolithography of periodic arrays of plasmonic nanoantennas onto transparent dielectric substrates. The introduction of a 10 nm thick ITO layer between an insulating substrate and the thermosensitive polymer film enables the fabrication of monodisperse plasmonic nanostructures of any arbitrarily defined shape, size, and spatial arrangement. Under this condition, we show the capability to control the lateral size down to the 60 nm range. These results show high-spatial-resolution performances that are competitive, with the figure typically observed in conventional t-SPL experiments, where nanoarrays are defined onto opaque semiconducting substrates. By changing the PMMA/MA resist thickness and consequently the etching time and the metal thickness, it was possible to extend the limit of the resolution of this technique under 60 nm without changing the process flow and the materials.

It is shown that plasmonic resonators, whose resonating frequency can be tuned on demand, are fabricated and analyzed by means of microtransmittance spectroscopy over a broad spectral range and with micrometric spatial resolution. The effective diameters of the obtained nanodiscs span from about 60 to 300 nm, and the gap between neighboring nanostructures is swept from about 125 to 350 nm so that the LSPR can range from 700 to 1350 nm. The high-quality factor of the LPR of nanostructures smaller than 150 nm paves the way for their fruitful exploitation in applications like biosensing, thermoplasmonic heating, and strong coupling. The versatility of this technique in terms of morphology and substrates can be further extended by changing the deposited materials and by coupling plasmonic oscillators with other types of optical resonators (like two-dimensional materials and quantum dots).
